# The Link Between the Gut Microbiome and Bone Metastasis

**DOI:** 10.3390/ijms252212086

**Published:** 2024-11-11

**Authors:** Aneta Sevcikova, Monika Martiniakova, Radoslav Omelka, Viola Stevurkova, Sona Ciernikova

**Affiliations:** 1 Department of Genetics, Cancer Research Institute, Biomedical Research Center of Slovak Academy of Sciences, 845 05 Bratislava, Slovakia; aneta.sevcikova@savba.sk (A.S.); viola.stevurkova@savba.sk (V.S.); 2 Department of Zoology and Anthropology, Faculty of Natural Sciences and Informatics, Constantine the Philosopher University in Nitra, 949 74 Nitra, Slovakia; mmartiniakova@ukf.sk; 3 Department of Botany and Genetics, Faculty of Natural Sciences and Informatics, Constantine the Philosopher University in Nitra, 949 74 Nitra, Slovakia; romelka@ukf.sk

**Keywords:** gut microbiome, cancer progression, bone metastasis, microbiota modulation

## Abstract

The gut microbiome is essential for regulating host metabolism, defending against pathogens, and shaping the host’s immune system. Mounting evidence highlights that disruption in gut microbial communities significantly impacts cancer development and treatment. Moreover, tumor-associated microbiota, along with its metabolites and toxins, may contribute to cancer progression by promoting epithelial-to-mesenchymal transition, angiogenesis, and metastatic spread to distant organs. Bones, in particular, are common sites for metastasis due to a rich supply of growth and neovascularization factors and extensive blood flow, especially affecting patients with thyroid, prostate, breast, lung, and kidney cancers, where bone metastases severely reduce the quality of life. While the involvement of the gut microbiome in bone metastasis formation is still being explored, proposed mechanisms suggest that intestinal dysbiosis may alter the bone microenvironment via the gut-immune-bone axis, fostering a premetastatic niche and immunosuppressive milieu suitable for cancer cell colonization. Disruption in the delicate balance of bone modeling and remodeling may further create a favorable environment for metastatic growth. This review focuses on the link between beneficial or dysbiotic microbiome composition and bone homeostasis, as well as the role of the microbiome in bone metastasis development. It also provides an overview of clinical trials evaluating the impact of gut microbial community structure on bone parameters across various conditions or health-related issues. Dietary interventions and microbiota modulation via probiotics, prebiotics, and fecal microbiota transplantation help support bone health and might offer promising strategies for addressing bone-related complications in cancer.

## 1. Introduction

Trillions of bacteria, viruses, fungi, archaea, and eukaryotic organisms inhabit the human gastrointestinal tract (GIT). The gut microbiome represents a complex community of all microorganisms residing in GIT, along with their genes and metabolic potential. Its composition changes throughout life due to various intrinsic and extrinsic factors, including gender, age, antibiotic use, dietary changes, physical activity, and many others [1,2]. Favorable microbiota composition is characterized by broad microbial diversity and high colonization resistance.

Recently, our understanding of the human gut microbiome about health and disease has rapidly expanded, primarily due to advances in sequencing technologies and bioinformatics methods. The importance of the microbiome in metabolism, nutrition, shaping the host’s immune system, and preventing numerous diseases is now well-established [3]. Mounting evidence highlights that disrupted gut microbiome composition significantly influences the onset and progression of cancer. Moreover, bacteria were found to be an integral part of the tumor microenvironment (TME), opening new possibilities for potential microbiome-based strategies. Ongoing research reveals that both the gut and tumor microbiomes can influence the response to anti-cancer therapies, especially chemotherapy and immunotherapy [4,5]. Tumor-associated bacteria can interact with immune cells and modulate their response to malignant cells. Numerous studies highlight the correlation between the favorable composition of gut microbial communities and improved therapy outcomes, leading to remission and prolonged patient survival. On the other hand, dysbiotic microbiome composition, characterized by a reduced diversity and high levels of pathogens, is associated with a diminished treatment response and poorer patient outcomes [6].

Alterations in gut microbial composition contribute to oncogenesis and might affect metastatic spreading to distant organs. As shown, microbial metabolites can not only initiate malignant cell transformation but also promote tumor progression and the formation of distant metastases [7]. The structured distribution of bacteria within tumors influences immune and epithelial cell activity [8], may regulate epithelial-to-mesenchymal transition (EMT)-related pathways [9], and alters the actin cytoskeleton in circulating tumor cells [10]. Thus, gut microbiota modulation offers a promising approach to enhancing cancer treatment efficacy [11,12] and metastatic spreading [13].

Several studies identified a close relationship between the immune and skeletal systems [14,15], and complex interactions were further explored through the field of “osteoimmunology” [16]. In 2018, Ohlson and Sjogren coined the term “osteomicrobiology” to describe a rapidly growing field of research focused on the impact of the gut microbiome on bone health. This interdisciplinary area bridges gastroenterology, immunology, microbiology, and bone physiology [17]. While previous studies have demonstrated that the gut microbiome, microbes, and immune cells are pivotal players in bone homeostasis, the research field of osteoimmunology evolved into a more complex area of osteomicrobiology. Analysis has progressed from animal studies to carefully designed clinical trials evaluating the effects of probiotics on bone health in postmenopausal women. An increasing number of trials aim to determine whether the gut microbiome can be targeted as a potential treatment for osteoporosis [17].

The current research is focused on revealing osteomicrobiology in the context of bone metastases. Osteomicrobiology describes the associations between the bone microenvironment and gut microbiome in health and disease [18]. However, how the gut microbiome affects bone metastasis development is still unknown [19]. The proposed mechanisms include processes in which the gut microbiome might promote bone metastases via stimulation of metastatic cancer cells, shaping the immune system, and affecting the bone microenvironment [20]. In this review, we summarize recent knowledge regarding the role of the gut microbiome in bone metastasis and assess the potential of microbiota modulation in bone metastatic spreading. Microbiota modulation by probiotics, prebiotics, and fecal microbiota transplantation (FMT) can contribute to restoring gut homeostasis, reducing inflammatory reactions, and might influence bone remodeling processes, potentially leading to improved outcomes for cancer patients.

## 2. Bone Metastasis Formation

Bone represents a common site for the development of metastases from lung, breast, or prostate tumors, but also kidney and thyroid cancer can metastasize to bone as well [21,22]. The phenomenon of bone metastases points to the importance of the bone microenvironment during cancer, as these metastases disrupt the balance between bone-forming osteoblasts and bone-resorbing osteoclasts, resulting in skeletal complications (e.g., bone pain, fractures, compression of the spinal cord, and disability) that adversely affect patient morbidity and quality of life [23,24]. During tumor metastasis, cancer cells first undergo EMT to dissociate from the primary tumors and enter the circulation [22,25]. Subsequently, these circulating tumor cells (CTCs) can extravasate from blood vessels and migrate to premetastatic niches (especially vascular and osteoblastic or endosteal niches present in the bone marrow), where they become disseminated tumor cells (DTCs) [23,26]. In general, DTCs interact with various cells in the bone microenvironment (e.g., endothelial cells, mesenchymal stromal cells, hematopoietic stem cells, and bone cells) [27]. Moreover, DTCs can either immediately colonize and proliferate or remain dormant before eventually developing into detectable metastases [28]. Only 0.02% of cancer cells that enter the bloodstream are thought to produce clinically observable metastases [29]. However, once metastases appear, they account for 90% of cancer-related deaths [30].

The bone marrow is the preferred metastatic site for DTCs due to its rich sources of neovascularization factors, growth factors, chemokines, and cytokines [24,25]. Furthermore, its endothelial and spongy structure and high blood supply pose a challenge for tumor cells to colonize and grow [31]. Therefore, bones rich in hematopoietically active red bone marrow and trabecular bone tissue, such as vertebrae, pelvis, ribs, and metaphysis of long bones, are the most common sites for skeletal metastases [32]. Current investigations have revealed the close involvement of bone remodeling during the progression of bone metastasis. Any disturbance in the dynamic balance between bone formation and resorption leads to diseases such as osteoporosis (excessive bone loss) and osteopetrosis (excessive bone formation), which create a more favorable bone metastasis environment for various primary tumor types [25,33]. It is widely recognized that osteoblasts and osteoclasts contribute to the regulation of DTCs in bone directly through interaction and indirectly through secreted factors. In general, bone formation has been shown to initiate and maintain tumor cell dormancy, whereas bone resorption reactivates DTCs. On the other hand, DTCs may hire osteoclast progenitors and elevate local osteoclast activity, potentially reactivating them from dormancy. This indicates that the ‘on-and-off dormancy switch’ of osteoblasts and osteoclasts could be influenced by DTCs [25,34,35]. It has been proven that inflammatory cytokines, primarily produced by osteoblasts during bone remodeling, play a dual role in regulating tumor cell dormancy. This may help explain why osteoblasts can both promote tumor cell growth (through the action of interleukin-1-beta: IL-1β and tumor necrosis factor-alpha: TNF-α) and induce tumor cell dormancy (by secreting factors such as leukemia inhibitory factor (LIF), CXC chemokine: CXCL12, and growth-arrest-specific protein 6 (Gas6)) in the bone marrow [36,37,38,39,40].

The growth of metastatic tumor cells in the bone niche is generally associated with either elevated production of molecules that disrupt bone homeostasis by stimulating osteolysis by osteoclasts or osteosclerosis by stimulating osteoblast activity [41]. Increased bone resorption, which is the result of excessive osteoclast activation, contributes to the formation of osteolytic bone metastasis. Tumor cells release factors that either directly (e.g., IL-8) or indirectly (e.g., parathyroid hormone-related peptide: PTHrP, IL-6) stimulate bone resorption. The aforementioned factors trigger the release of signaling molecules from the bone, including receptor activator of nuclear factor kappa beta ligand (RANKL), osteoprotegerin (OPG), transforming growth factor-beta (TGF-β), insulin-like growth factors (IGFs), platelet-derived growth factor (PDGF), and calcium (Ca), which in turn stimulate tumor proliferation and perpetuate the vicious cycle related to bone metastases. This cycle is reinforced by the secretion of tumor factors (dickkopf-related protein 1: DKK-1, sclerostin 1: SOST-1, BMP inhibitor noggin, and activin A) inhibiting osteoblast activity, which is manifested by damaged bone with decreased bone mineral density (BMD) [23,24,28,29]. Sclerotic bone metastases arise as a result of excessive osteoblast activation due to factors secreted by some tumors, including PDGF, IGF-1, bone morphogenetic proteins (BMPs), fibroblast growth factors (FGFs), and the activated Wnt pathway. In addition, endothelin-1 (ET-1) and OPG promote osteoblast growth due to inhibition of osteoclast activity. Conversely, osteoblasts respond to this stimulation by secreting IGF-1, IL-6, IL-8, FGFs, and TGF-β, all of which are able to stimulate tumor growth. Both tumor cells and osteoblasts secrete vascular endothelial growth factor (VEGF) to ensure vascularization. The interplay between tumor cells and osteoblasts creates a vicious circle supporting sclerotic lesions with impaired spongiosa [23,28,42,43]. Although the vast majority of bone metastases from solid tumors contain both osteolytic and osteoblastic components, one or the other phenotype predominates in specific bone metastatic cancers [41]. Osteolytic metastases occur in breast, kidney, and lung cancer as well as multiple myeloma, while prostate cancer is related to osteoblastic (sclerotic) lesions [28,44].

The modulation of immune responses is crucial in the progression and regulation of metastatic cancer. In the bone microenvironment, numerous immune cells (e.g., cytotoxic T cells: Tc cells, natural killer cells: NK cells, macrophages, regulatory T cells: Treg cells, myeloid-derived suppressor cells: MDSCs, and dendritic cells) contribute to the development of skeletal metastases [45]. Among these cells, Treg cells are particularly significant in the immune response to bone metastases, as they infiltrate tumor tissues and promote an immunosuppressive condition [46]. Targeting Tregs in cancer therapy is promising, as inhibition of Tregs may elevate response to radiotherapy and improve control of disease progression [23].

Primary tumor development followed by anti-cancer treatment can induce microbial dysbiosis by reducing intestinal colonization resistance and increasing the abundance of pathogenic microorganisms. As a result, an imbalanced gut microbiome might trigger a pro-inflammatory cascade and affect immune cells’ trafficking to the bone, where they prepare the microenvironment for the development of secondary bone metastasis. However, a deeper understanding of complex processes involved in bone metastasis development, where aggressive cancer cells leave primary tumors and migrate to distant bones, and the role of osteomicrobiology in this metastatic cascade remains essential for further investigation and precise characterization.

## 3. Microbiome and Cancer Progression

Maintaining intestinal homeostasis and mucosal barrier integrity represent the critical roles of the gut microbiome. Additionally, the impact of intestinal bacteria on the host immune system has been the subject of intense research [47]. Increasingly, studies documented associations between the gut microbiome, host organism, signaling pathways, and immune cells in cancer [7,48]. The improved concept “The Hallmarks of Cancer” summarized several characteristics that participate in tumor initiation and progression across the spectrum of different cancer types. As documented, polymorphic variability of oral, skin, lung, gut, tumor, and vaginal microbiomes might contribute to tumor initiation and progression via metastatic cascade promotion or affect the efficacy of anti-cancer therapies [49,50]. Battaglia et al. analyzed the presence of microbes in 4160 metastatic tumor samples using metagenomics and transcriptomics. The results showed that bacterial communities differed among anatomical sites, depended on primary tumor location, and correlated with therapeutic response [51].

A dysregulated microbiome affects the production of microbial metabolites and toxins, which might support EMT, angiogenesis, and tumor progression [7]. Lithocholic acid (LCA), a bacterial metabolite, can damage the intestinal barrier via produced reactive oxygen species, resulting in resistance to apoptosis and increased cell proliferation [52]. This metabolite might act as a tumor promoter implicated in colorectal cancer (CRC) metastases since LCA increased IL-8 expression by Erk1/2 activation and STAT3 suppression in CRC cells. IL-8 is considered a critical player in angiogenesis, and its blockade might inhibit tumor progression and angiogenesis [53]. Pathogenic microbe *Fusobacterium nucleatum* is implicated in CRC via the production of virulence factors involved in invasion, EMT, and disease progression [54]. Higher levels of *Fusobaterium nucleatum* in CRC tissue samples correlated with higher expression of disease progression markers, including E-cadherin, N-cadherin, and Nanog [55]. Similarly, the abundance of this pathogen was documented in stool and tumor samples from advanced-stage patients. *Fusobacterium nucleatum* promoted tumor-derived CCL20 expression in CRC tumors. In an animal model, the knockdown of CCL20 decreased *Fusobacterium*-induced CRC lung metastatic formation [56]. Also, *Bacteroides fragilis* toxin supports a loss of cell adhesion, leading to EMT [57]. Roje et al. revealed the underlying mechanisms by which gut microbiota facilitates the development of chemically-induced tumors and accelerates cancer progression in distant organs through microbiota-related metabolism of environmental carcinogens [58].

On the other hand, microbiota-derived metabolites such as short-chain fatty acids (SCFAs), including acetate, propionate, and butyrate, have anti-cancer properties [48] and might inhibit cancer stem cell proliferation, delay tumor development, and promote the expression of silenced tumor suppressor genes [59]. Butyrate decreased cell viability, migration, and invasion in breast cancer cell lines [60]. However, tumor microbiome-derived butyrate supported lung cancer metastases by inhibiting HDAC2 and increasing H3K27 acetylation at the H19 promoter, triggering M2 macrophage polarization. Depleted macrophages attenuated butyrate-induced metastasis formation [61]. Besides its anti-inflammatory effect [62], propionate promoted cell cycle arrest and apoptosis in lung cancer cell lines [63]. Moreover, sodium propionate treatment reduced glioblastoma cell migration and viability [64]. Changes in propionate metabolism supported metastatic and aggressive properties of breast and lung cancer cells [65]. *Propionibacterium*-related production of propionate and acetate had a protective effect via suppressed colon cancer cell proliferation and induced cancer cell death [66]. Furthermore, higher levels of SCFAs are positively correlated with response to immunotherapy in patients with metastatic or advanced solid tumors [67].

Probiotic bacteria can mitigate cancer progression via decreased inflammatory processes and elevated cancer cell apoptosis. Specific probiotic species can modulate the expression of oncogenes and tumor suppressor genes, impacting angiogenesis and metastatic formation [68]. The anti-cancer properties are also attributed to microbial structural components and bacterial metabolites [69]. A probiotic mixture containing *Lactobacillus rhamnosus* GG, viable *Escherichia coli* Nissle 1917, and heat-inactivated VSL#3 decreased liver tumor growth and downregulated proangiogenic genes. Probiotics also changed gut microbiome composition towards elevated levels of *Prevotella* and *Oscillibacter*, leading to the production of anti-inflammatory metabolites [70]. In a murine model, a capsaicin-rich diet had pro-metastatic potential by increasing pro-inflammatory cytokines, including IL-12, IL-6, TNF-α, and INF-γ. A higher dose of capsaicin might promote CRC cell metastasis to the liver by creating a metastatic niche. According to the findings, capsaicin diet dysregulated levels of mucin-related *Akkermansia* and *Muribaculaceae*, as well as other microorganisms involved in bile acid metabolism [71]. As recently shown, high-fat diet (HFD)-associated gut microbiota accelerates cancer progression by stimulating the production of polymorphonuclear myeloid-derived suppressor cells (PMN-MDSCs) through the activation of the mTORC1 signaling pathway in myeloid progenitor cells. Additionally, an increased prevalence of *Desulfovibrio* in the fecal microbiome of overweight breast cancer patients has been positively linked to cancer progression, along with elevated levels of fecal leucine and PMN-MDSCs [72].

The connection between osteomicrobiology and cancer progression is still in the early stages of research. However, findings indicate that a disrupted gut microbiome has a significant impact not only on primary tumors but also on spreading cancer cells to the liver, lung, brain, or bone due to favorable interaction with these distant organs. In the context of advanced cancer stage and bone metastasis, it is necessary to describe the complex underlying mechanisms by which microorganisms, microbial toxins, and altered immune system responses participate in the development of secondary bone cancer.

## 4. The Relationship Between Microbiome and Bone Homeostasis in Cancer

**Figure 1:**
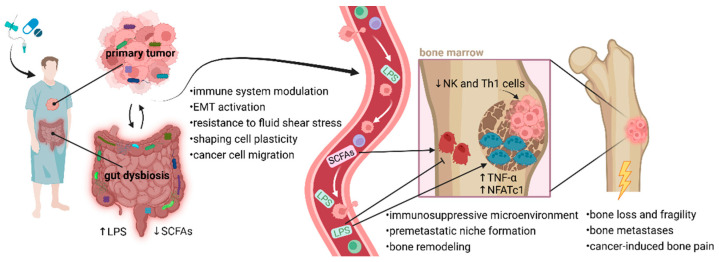
Proposed mechanisms linking dysbiotic gut and tumor microbiomes to bone metastasis. Anti-cancer therapies and pre-treatment with broad-spectrum antibiotics negatively affect the rigidity of the intestinal barrier, and changes in gut microbiome composition lead to a dysbalance between favorable bacteria and pathogens. Elevated gut permeability causes LPS translocation into circulation, which might promote cancer cell mobility. LPS or other bacterial metabolites interact with osteoclasts and osteoblasts, disrupting the balance between bone modeling and remodeling with accelerated metastasis building. The balance between osteoclast and osteoblast activity in bone is necessary for normal bone development. LPS support osteoclast function and activity in bone destruction via upregulated NFATc1 and TNF-α. Inappropriate osteoclast activity through bone degradation might prepare a favorable microenvironment (premetastatic niche) for early metastatic tumor cell colonization. Moreover, excessive osteoclast activity affects many pathophysiological processes in bones, leading to bone loss, fragility, fractures, and cancer-induced bone pain. An altered microbiome is associated with decreased production of favorable SCFAs, which play a role in osteoblastogenesis and bone health. Communication between the microbiome and bone metastases may occur through immune-mediated pathways. Considering the essential role of microbiome in immune system development, broad-spectrum antibiotics can affect the migration of NK and Th1 cells from the gut to the bone marrow. Reduced populations of Th1 and NK cells might support tumor growth within the bones. Typically, NK cells are developed in the bone marrow before migrating via the bloodstream to secondary lymphoid tissues, such as Peyer’s patches. Favorable microbiota interacts with NK cells, enhancing their activation and promoting cytolytic activity through the expression of granzyme B. Additionally, tumor-associated microbiota contributes to metastatic processes, including immune system modulation, EMT-related pathways, and matrix metalloproteinase regulation. Microorganisms residing in the TME can help cancer cells resist fluid shear stress by altering the actin cytoskeleton, which supports their survival in circulation and facilitates migration. Notably, a dysbiotic microbiome can modify the bone microenvironment, making it more conducive to cancer cell colonization. This allows tumor cells to adapt to the biochemical factors present in the premetastatic niche and subsequently initiate a metastatic cascade [18,19,78]. Abbreviations: EMT, epithelial-to-mesenchymal transition; LPS, lipopolysaccharides; NFATc1, nuclear factor of activated T cells, cytoplasmic 1; NK cells, natural killer cells; SCFAs, short-chain fatty acids; Th1 cells, type 1 T helper cell; TNF-α, tumor necrosis factor-alpha; TME, tumor microenvironment.

The gut microbiome can influence the bone microenvironment, and intestinal dysbiosis has been identified in individuals with several bone-related diseases, including osteoporosis, rheumatoid arthritis, osteoarthritis, bone cancer, or diabetes mellitus [73,74,75]. According to recent findings, the microbiome plays a critical role in regulating bone homeostasis via the gut-immune-bone axis, gut-brain axis, endocrine function, and host metabolism [75,76]. Microbiota-derived metabolites, such as SCFAs, can modulate immune responses and bone homeostasis [77]. Dysbiotic changes in gut microbiome composition result in altered levels of microbial metabolites, potentially promoting or inhibiting the cancer cell spreading and colonization within the bones (Figure 1).

### 4.1. The Role of the Microbiome in Bone Development

Maintaining bone health is essential for supporting the body’s construction, protecting vital organs, and serving as a reservoir for critical minerals. Ca and vitamin D are key players in structural bone integrity. A deficiency of vitamin D weakens bones, contributing to fracture development [79]. Furthermore, the interplay between the immune system and bone metabolism is crucial for optimal skeletal development. Since the gut microbiome positively affects immune cell maturation, the favorable composition of microbial communities contributes to skeletal health [80,81]. Moreover, microbiota-derived SCFAs increase Ca accessibility and Ca resorption and support bone mineralization and growth [82,83]. SCFAs are involved not only in energy metabolism but also affect osteoclast and osteoblast activity [79]. Mounting evidence from *in vivo* studies with ovariectomized mice documented a strong link between gut microbiome composition and bone metabolism, including BMD [84,85]. Despite some contraindicatory results, evidence highlights that gut microbiome composition is a BMD regulator via the immune system [86]. The altered gut microbiome is associated with the changes in bone mass and structure. In antibiotic-treated mice, femur bending strength was reduced, and treatment with ampicillin and neomycin led to the absence of B and T cells. The findings indicated that antibiotics altered the gut microbiome, resulting in depletions of members belonging to Bacteroidetes and enrichment of the Proteobacteria phylum [87]. Sjogren et al. showed that the frequency of CD4+ T cells and osteoclast precursor cells was reduced, together with decreased osteoclasts per bone surface in germ-free (GF) mice compared to conventionally raised mice. In addition, reduced expression levels of TNF-α and IL-1 and elevated IL-6 levels were detected in GF mice. The colonization of GF mice with normal microbiota led to increased bone mass. These findings indicated that the bone mass was affected by the impact of the gut microbiome composition on the immune system [88]. The colonization of sexually mature GF mice with microbiota obtained from conventionally specific pathogen-free mice elevated bone formation, but the results might depend on the duration of colonization. Moreover, data showed that broad-spectrum antibiotics or vancomycin inhibited bone formation, decreased serum levels of IGF-1, and changed gut microbiome in conventionally raised mice. IGF-1 is known for its role in skeletal growth. Antibiotics have been shown to decrease fecal levels of acetate and butyrate, but supplementation with SCFAs restored circulating IGF-1 levels [89]. Sex hormones play an important role in bone homeostasis, but their decreased level might affect the gut microbiome and contribute to bone loss [90,91]. GF mice were protected from trabecular bone loss induced by sex steroid deprivation. However, their recolonization restored the ability of sex steroid deficiency to induce bone loss. These results demonstrated that the microbiota is key in affecting cortical and trabecular bone volume in sex steroid deficiency [92]. Schwarzer et al. observed that bone growth parameters (cortical thickness, cortical and trabecular bone fractions, and femoral length) were reduced in GF mice but BMD did not change. Moreover, GF mice were 4% shorter in body length than wild-type mice and also had lower body weights [93].

**Figure 2:**
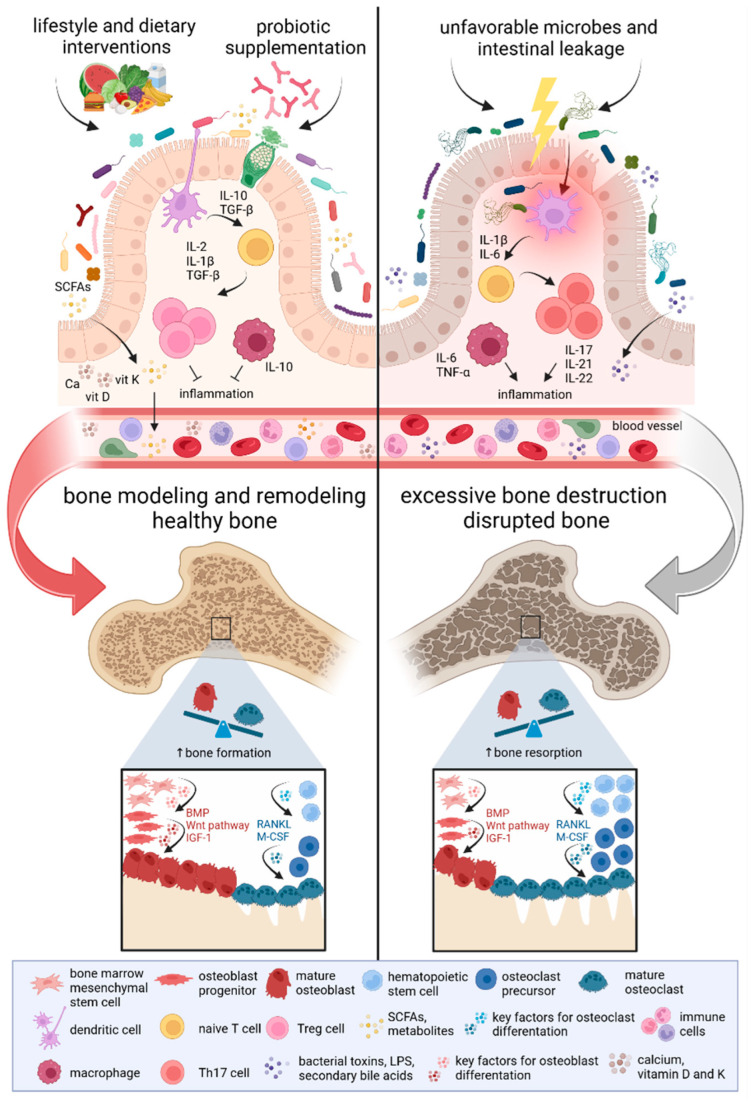
The impact of the gut microbiome on bone development. Gut microbiome composition regulates bone metabolism through the immune cascade along the microbiome-gut-bone axis. Studies focusing on the microbiome-bone crosstalk have shown that disruptions in the microbiome and the production of pro-inflammatory factors might promote bone resorption and lead to bone loss. However, microbiota modulation through lifestyle and dietary interventions, as well as probiotic supplementation, appears to support enhanced bone formation and increased bone mass. A healthy microbiome supports the processes of bone modeling and remodeling, maintaining the balance between bone resorption and formation. The production of microbiota-derived metabolites, such as SCFAs, prevents bone loss. Mounting evidence highlights the critical role of the gut microbiome in shaping the host’s immune system. An imbalance between Treg and Th17 immune cells has opposing effects on bone remodeling. Higher levels of Treg cells contribute to osteoblast differentiation and bone mass increase, whereas an increased amount of Th17 cells is associated with bone resorption and loss. Activation of key factors involved in osteoblastogenesis, including Wnt, BMP, and IGF-1, stimulates bone formation. Conversely, osteoclastogenesis is supported by essential factors such as RANKL and M-CSF [97,98,99]. Abbreviations: BMP, bone morphogenetic protein; IGF-1, insulin-like growth factor 1; IL, interleukin; LPS, lipopolysaccharides; M-CSF, macrophage colony-stimulating factor; RANKL, receptor activator of nuclear factor kappa beta ligand; SCFAs, short-chain fatty acids; TGF-β, transforming growth factor-beta; Th17 cells, T helper 17 cells; TNF-α, tumor necrosis factor-alpha; Treg cells, regulatory T cells.

Recent studies demonstrated a beneficial effect of favorable probiotic bacteria on bone development and metabolism (Figure 2). The supplementation with *Lactobacillus rhamnosus* GG increased bone mass, leading to elevated levels of Treg cells in eugonadic mice. Probiotic supplementation enhanced transcription of the gene encoding the enzyme butyryl-CoA:acetate CoA-transferase [94], which is responsible for butyrate production by intestinal lactate-utilizing bacteria [95,96]. An earlier study observed that *Lactobacillus rhamnosus* GG usage prevented sex steroid deficiency-induced bone resorption and bone loss in rodents [92]. Research data therefore suggests that the microbiota can influence bone metabolism, BMD, and bone mechanical properties.

### 4.2. Targeting the Microbiome in Bone Metastases and Treatment

Bone metastases remain an incurable condition, leading to significant health issues, including pain and fractures, adversely impacting patients’ quality of life. Consequently, palliative treatment is employed to alleviate pain and prevent bone metastasis-related complications [100,101]. Improved characterization of the metastatic environment in bones can help to develop novel therapeutic strategies. Immunotherapy might be considered as one of the few remaining options since bone serves as a secondary immune organ where bone marrow cells interact with immune cells [102,103,104]. However, cancer cells within the microenvironment create an immunosuppressive niche, contributing to decreased anti-cancer treatment response [105]. In contrast to primary tumors, bone metastases have lower immunogenicity, which causes decreased response to immunotherapy. Only limited data documented the effect of immunotherapies on bone metastases [102]. Patients with non-small cell lung cancer (NSCLC) and bone metastases did not respond to combined PD-1 blockade and anti-angiogenic therapy [106]. Overall survival was prolonged in patients with advanced renal cell carcinoma and bone metastases treated by nivolumab compared to patients treated by everolimus [107]. In a bone metastatic murine model, nivolumab injection protected against bone destruction by inhibiting TRAP+ osteoclast differentiation in the tumor-bearing femur [108]. Remarkably, Moseley et al. first reported skeletal adverse effects such as osteoporotic fractures and focal bone resorptive lesions related to PD-1, CTLA-4, or both therapies [109].

Recently, increasing data has shown a connection between the gut microbiome and therapy efficacy with a potential effect on bone health. The imbalanced microbiota, dominated by pathogenic species, could lead to reprogramming the bone microenvironment and creating a metastatic niche [110]. The gut microbiome can influence the process of bone remodeling through immune modulation, affecting the activity of both osteoclasts and osteoblasts and potentially fostering a microenvironment that allows tumor cells to thrive in bone tissue. The gut microbiome regulates the processes of activation, differentiation, and migration of T cells [111]. In addition, intestinal microorganisms influence NK cells, which have a critical role in anti-tumor immunity [112].

Broad-spectrum antibiotic administration (ampicillin, vancomycin, neomycin sulfate, and metronidazole) facilitated intra-bone tumor growth and osteolysis in the melanoma murine model [19]. Microbiota deficiency negatively affected the expansion of NK and Th1 cells in the bone marrow and Peyer’s patches, leading to enhanced tumor progression and bone loss via the gut-immune-bone axis [19]. A recent study by Dutta et al. demonstrated that antibiotic usage (vancomycin, neomycin, metronidazole, amphotericin, and ampicillin) damaged intestinal homeostasis, increased tumor growth, and promoted tumor cell dissemination in mice with triple-negative mammary cancer. The results showed a lower amount of *Lactobacillus*, *Oscillospira*, *Ruminococcus*, *Bifidobacterium*, and *Anaeroplasma* in the experimental model compared to controls. Antibiotic exposure resulted in elevated levels of gram-negative bacteria, while gram-positive taxa were diminished. Metagenome analysis revealed that the pathways associated with carbohydrate metabolism, energy metabolism, signal transduction, and metabolism of cofactors or vitamins positively correlated with a group of antibiotic-treated bone metastatic breast cancer mice. IL-4, an anti-inflammatory cytokine, showed decreased expression in the antibiotic-treated group. Moreover, the expression of inflammatory markers, including granulocyte-colony-stimulating factor (G-CSF) and matrix metalloproteinase-9 (MMP-9), was higher in serum samples from antibiotic-treated and untreated bone metastatic breast cancer mice compared to control mice. The study of bone marrow samples confirmed that macrophages, B cells, Tc, and helper T cells (Th cells) decreased in tumor-bearing and antibiotic-treated animals, which indicated that the immunosuppressive environment of bone marrow might be associated with successful bone metastasis development [110].

Uzelac et al. performed RNA sequencing of bone and tissue biopsies obtained from patients with metastatic castration-resistant prostate cancer. The results identified 31 differently presented bacterial species in bone metastases, 65 species in the liver, and 70 species in the lymph node samples. Bacterial species, including *Escherichia coli*, *Acinetobacter* spp., and *Mycobacterium leprae*, were presented in all cohorts [113]. Fernandes et al. confirmed that radiotherapy negatively impacts the presence of favorable gut bacteria. In castration-resistant prostate cancer patients with two or more bone metastases, the therapy based on radium-223 (Ra-223) led to gut microbiome changes, and the results showed that levels of *Clostridium coccoides*, *Clostridium leptum*, and *Bacteroides fragilis* were decreased in fecal samples after treatment [114]. In Longhua Hospital (China), Wenshen Zhuanggu Formula is used as complementary herbal medicine for the treatment of breast cancer bone metastases. The authors wanted to access the activity of 6 coumarins (psoralen, isopsoralen, bergapten, xanthotoxin, osthole, and imperatorin) in normal mice and a xenograft model of breast cancer bone metastases due to potentially affecting the pharmacokinetic properties of orally given formula in pathological conditions. Metastatic tumors negatively affected the pharmacokinetics and absorption of coumarins after oral administration. As concluded, gut microbiota composition might be responsible for decreased biotransformation of coumarin glycosides in breast cancer bone-metastatic mice. Therefore, coumarins might have reduced absorption in the bloodstream after oral administration of Wenshen Zhuanggu Formula [115].

A retrospective study on a large cohort of breast cancer patients showed accelerated bone metastasis progression in patients with untreated osteoporosis [116]. Wenhui et al. first assessed the composition of the gut microbiome in patients with breast cancer with/without bone metastases vs. normal controls. Their results confirmed a lower amount of *Megamonas*, *Clostridia*, *Akkermansia*, *Gemmiger*, and *Paraprevotella* while bacteria including *Lactobacillales*, *Bacilli*, *Veillonella*, *Streptococcus*, *Campylobacter*, *Epsilonproteobacteria*, *Acinetobacter*, *Pseudomonadales*, *Moraxellaceae*, and *Collinsella* had a higher prevalence in the presence of bone metastases. Moreover, the authors predicted the increased metabolic activity in the gut microbiome of breast cancer patients with bone metastasis in contrast to individuals without metastases. The detailed results revealed higher activity of biological processes, including secondary metabolite biosynthesis, transport and catabolism, steroid hormone biosynthesis, nitrogen metabolism, taurine/hypotaurine metabolism, and sulfur metabolism. Another comparison between patients with metastases and normal controls showed that pathways involved in lipid/nitrogen/folate/ascorbate/steroid hormone biosynthesis and bile acid metabolism and synthesis were upregulated in cancer patients [117]. The luminal A or B subtype of breast cancer is associated with the development of bone metastases [118].

Although the link between breast microbes and bone metastases remains poorly understood, Naik et al. provided an excellent review of the role of breast and gut microbiomes in the development of bone metastases. Microbiomes might affect EMT, metabolism of steroid hormones, immunity, bone remodeling, and secretion of metabolites that change TME and contribute to potential metastases from breast tumors [78].

### 4.3. The Impact of Microbiota Modulation on Bone Health

Combining immunotherapy with immune checkpoint inhibitors and strategies for microbiota modulation could improve cancer treatment efficacy [119,120,121,122]. Gut microbiome composition influences degenerative bone-related diseases characterized by reduced bone mass and damaged bone microarchitecture [99,123]. Recent studies propose the potential of a microbiome-based approach for maintaining bone health and potentially managing bone metastasis formation.

Various diets and nutritional strategies successfully preserve bone health [97], and numerous ongoing clinical trials aim to comprehensively evaluate the associations between microbiome composition and bone parameters (Table 1).

An orally administered diet enriched with polyamine-rich *Saccharomyces cerevisiae* S631 prevented osteoclastic activation in the ovariectomized murine model [124]. Also, there is a connection between iron metabolism and bone health. Excess iron might be responsible for bone loss [125]. Therefore, patients with sickle cell disease, thalassemia, and hereditary hemochromatosis had a higher prevalence of fractures and osteoporosis [126]. The exposure to high fat and high sugar within a Western-style diet led to polarized bone marrow-derived macrophages toward their inflammatory state and subsequently induced gut dysbiosis [127]. The extent to which modulating the macrophage response through the restoration of the gut microbiome may protect against the development of a premetastatic microenvironment and subsequent metastatic formation has not been thoroughly investigated yet [128]. The Mediterranean diet (MD), considered one of the healthiest diets, is associated with changes in the gut microbiome with an increase in *Bifidobacterium animalis* in the Spanish population [129]. Takimoto et al. observed that a higher level of favorable *Bifidobacterium* improved BMD via decreased bone resorption, as reflected by a reduced level of the bone resorption marker tartrate-resistant acid phosphatase isoform 5b (TRACP-5b) [130]. However, some studies documented the contraindicatory effect of MD on bone health [131]. MD enriched with virgin olive oil for 24 months had a protective effect on bone through elevated levels of bone formation markers osteocalcin and procollagen type I N-propeptide in elderly men [132]. The adherence to MD decreased hip fracture incidence in adult participants [133]. On the contrary, Feart et al. noted that MD did not correlate with a lower risk of fractures at any site in French older persons. When authors focused on diet components, the results showed that greater fruit consumption was associated with a higher risk of hip fractures, while low consumption of dairy products correlated with a doubled risk of wrist fractures [134]. In adult Greek women, MD did not significantly affect bone mass maintenance. Conversely, dietary compounds similar to MD, including fish and olive oil, and their higher intake were positively related to total body bone mineral content (BMC) and lumbar spine BMD [135].

Probiotic supplementation, in particular, has been shown to boost the activity of immune effector T cells while reducing the activity of immune suppressor T cells [20]. Supplementation with *Lactobacillus reuteri* NCIMB 30,242 increased serum levels of 25-hydroxyvitamin D and reduced the risk of osteoporosis in healthy hypercholesterolemic participants [136]. Engineered probiotic strain *Lactococcus lactis* is capable of expressing a fusion protein of Fms-like tyrosine kinase 3 ligand and co-stimulator OX40 ligand (FOLactis), which might effectively activate immune cells (dendritic and T cells). The injection of FOLactis into the bone marrow had a suppressive effect on tumor growth in established murine models of bone metastasis. The authors demonstrated that FOLactis increased mature dendritic cells and CD8+ T cells and prolonged the survival of tumor-bearing mice [137].

Certain microbial metabolites produced by the gut microbiome might have an impact on cancer and bone cells, including processes implicated in forming specific microenvironments for cancer cells [78]. Supplementation with SCFAs increased bone mass while decreasing trabecular separation in C57BL/6J mice. Both propionate and butyrate showed a protective effect on bone mass via inhibited osteoclast differentiation and decreased bone resorption. However, the treatment with acetate did not reduce the osteoclast numbers [77]. In vitro experiments demonstrated that butyrate inhibited osteoclast formation and resorption and promoted osteogenic differentiation of mesenchymal stromal cells. Subsequent in vivo experiments showed reduced levels of pro-inflammatory cytokine IL-6 in butyrate-treated mice after osteotomy. On the other hand, antibiotic treatment reduced the cecal level of SCFAs, changed gut microbiome composition, and increased the level of pro-inflammatory markers (TNFα, IL-6, IL-17a, and IL-17f). Moreover, antibiotics delayed bone healing in the animal osteotomy model, while butyrate supplementation did not significantly affect this process [138]. Although SCFAs are crucial regulators of bone homeostasis, their excessive intake might negatively affect host organisms [139]. The pathogenic strain *Escherichia coli* O157 produces a harmful substance known as Shiga toxin. This toxin can bind to globotriaosylceramide, the expression of which was enhanced in butyrate presence. Infection with *Escherichia coli* O157, along with supplementation of a high-fiber diet, resulted in increased butyrate production and elevated expression of globotriaosylceramide, leading to an intensive binding of Shiga toxin in a murine model. The high fiber diet also changed gut microbiome composition with reduced native *Escherichia* spp. and increased Shiga toxin-producing *Escherichia coli*. Therefore, the individual diet habits and ability of gut microbes to produce butyrate might affect the development of infection after *Escherichia coli* O157:H7 ingestion [140]. A new strategy in the prevention of cancer progression might be the modulation of gut microbiome via prebiotics. Polyphenols and plant-derived phytochemicals act as prebiotics, and the studies assessed their effect on tumor growth and metastases [141]. Castillo-Pichardo et al. tested the impact of combined supplementation with resveratrol, quercetin, and catechin (5 mg/kg each polyphenol) on mammary tumor progression in a murine model with an injected bone metastatic variant of MDA-MB-435 (ER−) cancer cells. The results revealed reduced mammary tumor growth by the aforementioned dietary polyphenols. Moreover, only 2/8 mice had metastatic foci in their femurs after oral gavage of combined polyphenols [142].

An experimental study showed that prebiotic fibers increased mineral absorption and BMD in the weanling rat model [143]. In a human study, a prebiotic mixture of short and long degrees of polymerization inulin-type fructan products promoted Ca absorption and bone mineralization in pubertal adolescents [144]. Another strategy for gut microbiota modulation is FMT. This method allows the transfer of microbiota from precisely selected donors to the intestinal tract of corresponding recipients to reshape microbiome composition and restore microbial diversity. Zhang et al. proposed that microbiota modulation via FMT might play a role in the regulation of bone metabolism and maintaining a balance between bone formation and resorption by repairing the mucosal barrier [145]. Transfer of feces from healthy C57BL/6 mice as donors prevented bone loss and osteoclastogenesis in ovariectomy-induced recipient mice. Ovariectomy altered the gut microbiome with a decreased amount of *Bacteroidia* and increased levels of *Melainabacteria*, while feces transfer restored this imbalance. Moreover, the results revealed a higher level of fecal SCFAs and increased expressions of tight junction proteins, including Zonula occludens protein 1 (ZO-1) and Occludin after FMT [146].

Modulating the gut microbiota through the aforementioned strategies promotes the growth of favorable gut microbes that help to eliminate harmful pathogens by enhancing both adaptive and innate immune responses while also supporting the integrity of the intestinal barrier. In the context of bone metastasis, restoring an intestinal balance is supposed to influence the bone microenvironment by creating specific conditions not suitable for the nidation of metastatic cancer cells.

## 5. Conclusions and Future Directions

Beneficial gut microorganisms exert a protective effect against pathogens, produce various metabolites and vitamins, and interact with the immune system, thereby shaping immune responses in the human body.

The connection between the changes in gut microbiome composition and severe diseases has garnered more attention. Mounting evidence highlights the impact of microbial communities on both cancer development and treatment outcomes. Moreover, the gut microbiome plays an emerging role in cancer progression and bone metastasis spreading by influencing immune responses, producing microbiota-derived metabolites, and affecting cancer cell features. According to the findings, several pathogenic microbes, including *Escherichia coli*, *Fusobacterium nucleatum*, *Bacteroides fragilis*, or *Helicobacter pylori*, along with their harmful metabolites, possess a pro-metastatic potential that facilitates the metastatic cascade of aggressive cancer cells, promoting their seeding, survival, and proliferation in secondary organs with a supportive microenvironment. On the other hand, probiotic *Lactobacillus*, *Bifidobacterium*, and microbiota-derived SCFAs can attenuate cancer progression, prevent the formation of metastatic lesions in distant organs, and prolong survival.

Recent findings uncovered several mechanisms by which bacteria residing in TME interact with cancer cells in a way that facilitates their invasive and migratory properties. Gut bacteria may help cancer cells adapt to new environments in metastatic niches, such as bone, by providing necessary signals for survival and proliferation. However, multifaceted challenges are faced when attempting to modulate the microbiota to improve outcomes in bone metastasis. First, further research is required to evaluate the specific mechanisms by which the gut microbiome affects bone health and cancer progression. Additionally, detecting and studying low-abundance microorganisms that might be involved in bone metastasis remains a technical challenge.

In conclusion, microbiome composition is a key player in maintaining bone health. A decreased bone formation coupled with increased bone resorption contributes to establishing a microenvironment conducive to bone metastasis development through immune-related mechanisms. The characterization of the gut microbial profile in patients with cancer and bone metastases, along with a deeper understanding of outlined correlations, may help to develop diagnostic, prognostic, and therapeutic tools for personalized treatment strategies. Targeting the microbiome-gut-bone signaling pathways, including those affecting SCFA production, may enhance patient outcomes and improve the management of bone-related complications in cancer care.

## Figures and Tables

**Table 1:** The list of ongoing clinical trials characterizing gut microbiome composition, bone health, or bone/body parameters under several conditions or health-related issues. Moreover, several trials evaluated the effect of different interventions on skeletal health and microbial diversity (according to https://ClinicalTrials.gov/, accessed on 22 October 2024).

ClinicalTrials.gov ID	Study Type	Conditions/ Health-Related Issues	Purpose	Subjects (n)	Intervention/Treatment	Study Status
NCT05348694	An interventional randomized placebo-controlled double-blinded study parallel assignment	Postmenopausal osteopenia/ bone loss/ age-related sarcopenia/ glucose metabolism disorders/ age-related cognitive decline	To study the impact of probiotics on BMD and assess the shifts in gut microbiome composition and changes in serum levels of bone turnover markers, focusing on Treg cells in blood samples	160 participants	Women will be supplemented with placebo or probiotic capsules containing *Clostridium butyricum*, *Clostridium beijerinckii*, *Anaerobutyricum hallii*, and *Bifidobacterium infantis*, plus chicory inulin and magnesium stearate (2 per day).	Active, not recruiting
NCT06265246	An interventional randomized non-label study parallel assignment	Osteoporosis/ obesity	To examine the effect of milk and fermented milk products on the gut microbiome, BMD, femoral neck BMD, serum levels of osteocalcin, procollagen, N-terminal telopeptide of type 1 collagen, serum C-terminal telopeptide of type 1 collagen, and IGF-1	99 participants	Participants will be divided into groups supplemented with habitual diet/habitual diet + 1.5 servings of milk/habitual diet + 2 servings of yogurt.	Recruiting
NCT06050018	An interventional randomized open-label study crossover assignment	Menopause	To analyze how milk influences gut microbiome diversity, pro-inflammatory cytokines, and bone remodeling through serum C-telopeptide, osteocalcin, and parathyroid hormone measurements.	15 participants	The diet will include milk and dairy products for 4 weeks, or an alternative protein source without any milk or dairy products for 4 weeks.	Not yet recruiting
NCT05548517	An interventional randomized open-label study parallel assignment	Weight loss/ time-restricted feeding/ bone loss	To monitor weight loss, BMD, bone volume, blood pressure, and microbiome composition	48 participants	Analyzed subjects will adhere to time-restricted eating and daily calorie restriction or daily calorie restriction alone.	Active, not recruiting
NCT05213780	An observational prospective study	Child development	To evaluate the relationship between maternal soy intake, the infant gut microbiome, and a child’s bone development while examining BMD, dynamic bone formation parameters, and the mRNA expression of bone markers.	240 participants	No intervention is involved.	Recruiting
NCT05160675	An observational prospective study	Healthy state	To determine gut microbiome composition, its function, bone health, and bone development from birth to 3 years and analyze dietary intakes, health outcomes, urinary markers of bone metabolism, and breast milk composition	2760 participants	No intervention is involved.	Active, not recruiting
NCT06519877	An interventional randomized study crossover assignment	Aging	To investigate the impact of soluble corn fiber on the gut microbiome, intestinal Ca absorption, and biochemical markers of bone turnover	30 participants	The intervention will include soluble corn fiber or placebo (maltodextrin) for 4 weeks.	Not yet recruiting
NCT06324084	An observational cross-sectional case-control study	Osteoporosis/ bone fracture/ cortisol excess	To evaluate the prevalence of hidden hypercortisolism in osteoporotic/osteopenic patients and investigate serum markers related to bone health, such as osteocalcin, bone alkaline phosphatase, and the amino-terminal propeptide of type 1 procollagen, along with potential new serum biomarkers. Moreover, the study aims to analyze the composition of the gut microbiome.	1500 participants	No intervention is involved.	Recruiting
NCT06389539	An interventional randomized study parallel assignment	Osteoporosis/ inflammation/ aging	To measure BMD of the lumbar spine, integral and vertebral trabecular BMD, and biochemical marker of bone resorption/formation and analyze gut microbiome function	220 participants	Subjects will be daily supplemented with SBD111 medical food (oligofructose, dried berry powder, *Pseudomonas fluorescens*, *Lactobacillus brevis*, *Leuconostoc mesenteroides*, *Lactobacillus plantarum*, and *Pichia kudriavzevii*) or placebo capsules over 18 months.	Not yet recruiting
NCT06133530	An interventional randomized study parallel assignment	Healthy state	To characterize bone formation/resorption markers, fecal calprotectin, inflammation markers in blood, fecal SCFAs, and gut microbiome	26 participants	Prebiotic human milk oligosaccharide or placebo (maltose) will be applied orally.	Enrolling by invitation
NCT04730622	An observational prospective study	Osteoporotic fracture of femur/ Osteoarthritis	To study IGF-1 serum level, serum markers of bone metabolism, and inflammatory markers and characterize gut microbial diversity	100 participants	Patients who are candidates for hip replacement surgery will be enrolled.	Recruiting
NCT05623098	An observational prospective study	Sepsis	To investigate whether nutrients containing dietary fiber affect bone metabolic markers, gut microbiome, SCFAs, and IL-6, TNF-α, and procalcitonin levels	2 participants	Subjects will receive dietary fiber enteral nutrition.	Active, not recruiting
NCT02822378	An interventional randomized open-label study parallel assignment	Postmenopausal osteoporosis	To determine BMD of the lumbar spine, total hip, and femoral neck and evaluate gut microbiome changes and serum concentrations of bone signaling markers (i.e., IGF-1, RANKL, osteoprotegerin, and sclerostin) before and after the intervention	322 participants	All subjects will be randomized into 50 g or 100 g dried plum group supplementation together with Ca and vitamin D for 52 weeks.	Active, not recruiting
NCT03455868	An observational prospective study	Morbid Obesity/ diabetes mellitus type 2	To evaluate the impact of the bariatric procedure on BMD at the lumbar spine, hip, tibia, and radius and assess changes in serum bone formation markers, hormones involved in bone metabolism, and gut microbiome composition before and after surgery	105 participants 20 controls	Patients with type 2 diabetes and obesity will undergo bariatric surgery (sleeve gastrectomy, Roux-in-Y gastric bypass, or biliopancreatic diversion with duodenal switch).	Recruiting
NCT06323538	An observational multi-center study	Cardiovascular diseases/diabetes mellitus type 2	To determine the health benefits/risks of diets, measure body composition, bone health via hormones involved in bone metabolism, cardiovascular risk factors, and diabetes risk, and analyze gut microbiome	6000 participants	Intervention will consist of vegan diet/vegetarian diet/pescetarian diet/mixed diet.	Not yet recruiting
NCT05802121	An interventional randomized study parallel assignment	Metastatic, castration-sensitive prostate cancer/ metabolic syndrome/ obesity/ cardiovascular morbidity	To evaluate whether oral acetate can improve the amount of *Akkermansia muciniphila* in the microbiome, metabolic parameters, and bone health via vitamin K2 levels	30 participants	Participants will take 6 enteric slow-release acetate capsules vs. 6 placebo capsules per day for 3 months.	Not yet recruiting
NCT02916862	An interventional randomized study parallel assignment	Osteoporosis	To analyze body fat, BMD, vitamin D status, serum levels of Ca, phosphate, alkaline phosphatase, osteocalcin, IGF-1, and creatinine, and characterize gut microbiome with a diversity of bacterial communities	240 participants	Subjects will consume soluble corn fiber/soluble corn fiber + Ca/placebo/placebo + Ca twice a day for 1 year.	Recruiting
NCT05332626	An interventional randomized, double-blinded placebo-controlled study parallel assignment	Postmenopausal osteoporosis	To measure the concentration of Ca, BMD, body parameters, biomarkers of bone turnover, and determine gene polymorphism	60 participants	The intervention will contain probiotic *Lactobacillus acidophilus* UALa-01 or placebo, which will be administered daily for 12 weeks.	Active, not recruiting
NCT03518268	An interventional randomized study parallel assignment	Breast cancer/ osteoporosis/ osteopenia	To assess changes in bone formation markers, IL-17, and TNF-α	40 participants	Probiotic intervention Vivomixx with 8 bacterial strains: *Lactobacillus*, *Bifidobacterium*, and *Streptococcus* vs. placebo will be administered for 6 months.	Unknown status
NCT06375668	An interventional randomized study parallel assignment	Postmenopausal osteoporosis,	To identify the effect of the intervention on BMD, levels of Ca, phosphorus, alkaline phosphatase, and vitamin D and determine types of stool and the number of bowel movements or adverse events	170 participants	A probiotic supplement will contain *Lactobacillus plantarum* and *Lactobacillus paracasei* while placebo capsules contain maltodextrin. All participants will receive 1 capsule per day for 1 year.	Active, not recruiting
NCT05350579	An interventional randomized open-label study parallel assignment	Osteoporosis/ inflammation	To reveal changes in serum markers of bone formation and resorption, as well as inflammatory cytokines, while also analyzing gut microbiome composition and SCFAs in fecal samples	33 participants	Participants will receive yogurt intervention containing 2 probiotic strains of *Streptococcus thermophilus* and *Lactobacillus bulgaricus.*	Terminated

Abbreviations: BMD, bone mineral density; Ca, calcium; IGF-1, insulin-like growth factor 1; IL-6/IL-17, interleukin-6/interleukin-17; mRNA, messenger ribonucleic acid; RANKL, receptor activator of nuclear factor kappa beta ligand; SCFAs, short-chain fatty acids; TNF-α, tumor necrosis factor-alpha.

## List of Abbreviations

BMC	bone mineral content
BMD	bone mineral density
BMPs	bone morphogenetic proteins
Ca	calcium
CRC	colorectal cancer
CTCs	circulating tumor cells
CXCL12	CXC chemokine
DKK-1	dickkopf-related protein 1
DTCs	disseminated tumor cells
EMT	epithelial-to-mesenchymal transition
ET-1	endothelin-1
FGFs	fibroblast growth factors
FMT	fecal microbiota transplantation
FOLactis	Fms-like tyrosine kinase 3 ligand and co-stimulator OX40 ligand
Gas6	growth-arrest-specific protein 6
G-CSF	granulocyte-colony-stimulating factor
GF	germ-free
GIT	gastrointestinal tract
HFD	high-fat diet
IGF-1	insulin-like growth factor 1
IGFs	insulin-like growth factors
IL	interleukin
IL-1β	interleukin-1-beta
IL-6/IL-17	interleukin-6/interleukin-17
LCA	lithocholic acid
LIF	leukemia inhibitory factor
LPS	lipopolysaccharides
M-CSF	macrophage colony-stimulating factor
MD	Mediterranean diet
MDSCs	myeloid-derived suppressor cells
MMP-9	matrix metalloproteinase-9
mRNA	messenger ribonucleic acid
NFATc1	nuclear factor of activated T cells, cytoplasmic 1
NK cells	natural killer cells
NSCLC	non-small cell lung cancer
OPG	osteoprotegerin
PDGF	platelet-derived growth factor
PMN-MDSCs	polymorphonuclear myeloid-derived suppressor cells
PTHrP	parathyroid hormone-related peptide
Ra-223	radium-223
RANKL	receptor activator of nuclear factor kappa beta ligand
SCFAs	short-chain fatty acids
SOST-1	sclerostin 1
Tc cells	cytotoxic T cells
Th cells	helper T cells
Th1 cells	type 1 T helper cells
Th17 cells	T helper 17 cells
TGF-β	transforming growth factor-beta
TME	tumor microenvironment
TNF-α	tumor necrosis factor-alpha
TRACP-5b	tartrate-resistant acid phosphatase isoform 5b
Treg cells	regulatory T cells
VEGF	vascular endothelial growth factor
ZO-1	Zonula occludens protein 1
